# Mapping Nurse Practitioners' Scope of Practice Laws: A Resource for Evaluating Pre-Exposure Prophylaxis Prescriptions

**DOI:** 10.1089/heq.2021.0113

**Published:** 2022-01-20

**Authors:** Sheila Salvant Valentine, Neal Carnes, Joseph Caldwell, Deborah Gelaude, Raekiela Taylor

**Affiliations:** Division of HIV Prevention, National Center for HIV, Viral Hepatitis, STD and TB Prevention, Centers for Disease Control and Prevention, Atlanta, Georgia, USA.

**Keywords:** HIV, PrEP, nurse practitioners, scope of practice

## Abstract

**Context:** Reducing the number of new HIV infections will require addressing barriers to HIV pre-exposure prophylaxis (PrEP) access and uptake. Nurse practitioners (NPs) may help increase PrEP access and uptake. State scope of practice laws determines NPs' ability to work independently and their authority to prescribe PrEP, a legend nonscheduled medication.

**Methods:** This analysis applied legal epidemiology methods to analyze the laws of the 50 states and the District of Columbia that govern NPs' scope of practice as they may apply to prescribing legend nonscheduled medications. These laws were extracted from Westlaw Next between April and June 2019.

**Results:** As of June 8, 2019, 17 states had laws that allowed NPs to both practice independently and prescribe legend nonscheduled drugs without restriction.

**Conclusion:** The role that state scope of practice laws plays in potentially limiting NPs' ability to prescribe PrEP should be considered. Increasing PrEP access and uptake is essential in reaching national HIV prevention goals. This analysis can inform further studies and polices on barriers to PrEP access and uptake.

## Introduction

The U.S. Department of Health and Human Services' Ending the HIV Epidemic in the United States (EHE) initiative requires addressing barriers to pre-exposure prophylaxis (PrEP) access and uptake.^1^ PrEP is medicine people at risk for HIV take to prevent getting HIV from sex or injection drug use. When taken as prescribed, PrEP is highly effective for preventing HIV.^2^ In 2015, an estimated 1.1 million adults, across three transmission risk groups, had indications for PrEP use.^3^ The three transmission risk groups examined were gay, bisexual, and other men who have sex with men; persons who inject drugs; and heterosexually active adults. Black people in all transmission risk groups; gay, bisexual, and other men who have sex with men are priorities for PrEP access and uptake.^3,4^

Primary care settings are encouraged to provide the clinical aspects of PrEP and primary care is increasingly staffed by nurse practitioners (NPs).^5,6^ NPs with independent scope of practice are more likely to prescribe PrEP than NPs with restricted scope of practice.^6^ Therefore, NPs can play an important role in increasing PrEP access and uptake, especially with independent scope of practice.^6^

NPs' ability to work independently, and prescribe PrEP, a legend nonscheduled drug (i.e., requires a prescription, yet poses little to no risk for dependency or abuse), is determined by state scope of practice laws.^7^ Fostering an environment where NPs can prescribe PrEP and perform appropriate patient follow-up activities can help increase PrEP access and uptake.^6^ This is particularly true for the communities receiving EHE resources from the Centers for Disease Control and Prevention, as a critical component of the funding is to increase PrEP prescriptions among persons with indications for PrEP.^8^

This analysis applied legal epidemiology methods to characterize state laws governing NPs' scope of practice, as they apply to legend nonscheduled drug prescribing, to inform further studies and policies addressing barriers to PrEP access and uptake.

## Methods

The research team developed a cross-sectional legal data set of 2019 statutes and regulations (laws) for all 50 states and the District of Columbia (D.C.) (jurisdictions), which addressed NPs' scope of practice, with a focus on prescriptive authority for nonscheduled legend drugs. The research team applied content analysis techniques using a standardized questionnaire to the relevant laws.

The team researched the laws of 10 states to define the project's scope, develop search terms, as well as define inclusion and exclusion criteria that determined which laws answered the research questions (Please see Appendix A1 for questions). The 10 states were chosen based on the NPs' scope of practice classification designed by the National Conference of State Legislatures (NCSL) and the Association of State and Territorial Health Officials (ASTHO) (scopeofpracticepolicy.org). This classification was chosen to be consistent with and build on previous research.

We chose the 10 states based on different geographic locations, and from the three categories included in the classification. Four states were randomly selected from the most restrictive category, three from the less restrictive category, and three from the least restrictive category. Inclusion criteria included scope of practice in terms of diagnostic, and treatment planning authority, supervision requirements, and authority to prescribe legend drugs. Exclusion criteria included requirements to obtain NP license or certification (except transition period required for full practice), authority to prescribe controlled substances, dispensing medication, and temporary certification.

Between April and June 2019, the team used WestlawNext, a legal database, to research the laws with the search terms: “adv: TI,PR,TE((scope/s practice) authori! licens! & ‘nurse practitioner’ A.N.P.).” Keyword searches were supplemented by reviewing the table of contents of each law (i.e., articles, subchapters, chapters, subtitles, and titles) to capture any other relevant law.

As a quality control measure to identify discrepancies in the research, the team conducted 100% redundant research where three members of the team (one original researcher and one of two redundant researchers) independently identified and recorded relevant laws' citations for each of the 51 jurisdictions. The team reviewed the results using the inclusion and exclusion criteria to identify and resolve divergences in results between the original and redundant searches.

After an initial review of included laws, the team drafted a questionnaire used to code the laws based on constructs and important features of the laws. For example, whether the law required NPs to have a relationship with a physician to practice. Subject matter experts reviewed the questionnaire before application. The questionnaire allowed the team to elicit and code objective observations and not subjective interpretations of the law. Coded law responses were entered on individual sheets for each of the 51 jurisdictions.

For quality control, the team conducted 100% redundant coding where six members of the team (one original researcher and one of five redundant researchers for each law) independently coded the relevant laws for each of the 51 jurisdictions. While coding, the team refined the project's scope to focus on NPs' prescribing authority for legend nonscheduled drugs. When coding was compared during quality control, there were no discrepancies to be resolved.

## Results

**FIG. 1.**
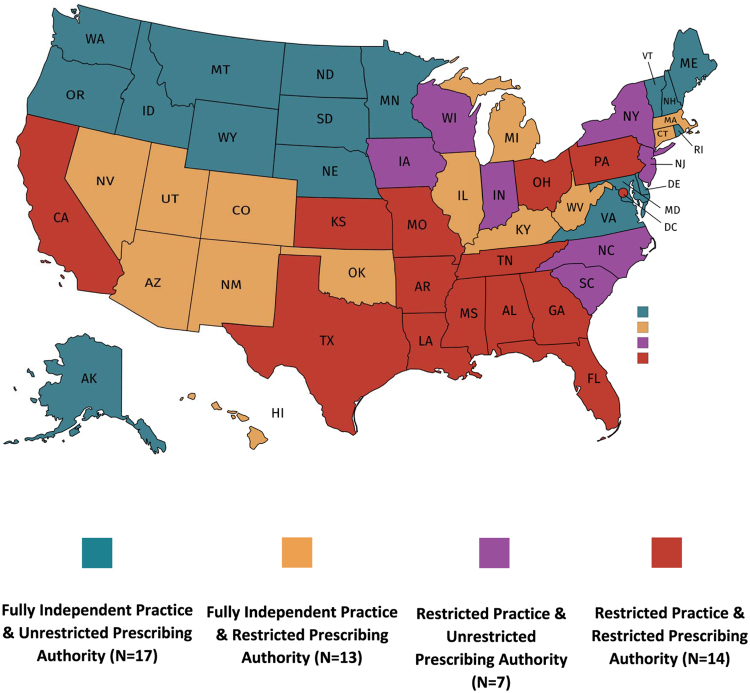
Map of nurse practitioner scope of practice and prescribing authority in 2019.

All 51 jurisdictions had laws relevant to NPs' scope of practice, including their ability to examine, diagnose, and treat (See Fig. 1 for a map). The laws in 21 jurisdictions did not allow NPs to practice independently. The laws in 30 states allowed NPs to practice independently. Of these 30 states, 27 had laws that allowed NPs to practice independently without restrictions; 2 had laws that allowed NPs to practice independently if the NP was not prescribing medications; and 1 allowed NPs to practice independently if not prescribing scheduled drugs (Supplementary Table S1).

Of the 30 states that allowed NPs to practice independently, 20 allowed independent practice from day 1, as opposed to after a transition period. Ten of the 30 states required NPs to practice under an oversight relationship during a transition period that varied between 1040 hours and 5 years. These 10 states required NPs to have a formal relationship, during the transition period, either with a physician, other NPs who have already gained independent scope of practice, or with a medical director (Supplementary Table S2). Of the 21 jurisdictions that did not allow NPs to practice independently, the type of on-going relationship required was either not described, or required working with a physician, podiatrist, medical staff, or dentist.

For the 31 jurisdictions that required NPs to have an oversight relationship (always or just during a transition period), the terminology in the laws describing the type of relationship required varied widely between states. The nonexhaustive list of terms used included “collaborative agreement,” “standard protocols,” “supervision,” “supervisory protocol,” and “practice agreement.” Five states used the term “supervision,” with two states qualifying the supervision as “direct,” and one as “not continuous and not constant.” Although the specifics and terminology varied, the general oversight requirement results in restricted scope of practice, including prescribing authority, for NPs practicing in these 31 jurisdictions.

No jurisdiction had laws explicitly addressing NPs' ability to prescribe PrEP. Twenty-seven jurisdictions had laws limiting NPs' prescribing authority in general. Two of the 27 jurisdictions that limited prescribing authority restricted NPs' authority regarding all drugs, scheduled and nonscheduled, based on written agreement with a collaborative physician, which would include PrEP (Supplementary Table S3).

Furthermore, 13 jurisdictions where NPs can practice independently limited NPs' prescribing authority, of which one limited prescribing authority regarding all drugs (Oklahoma); 14 jurisdictions where NPs cannot practice independently limited NPs' prescribing authority, of which one state limited prescribing authority regarding all drugs (Pennsylvania) (Supplementary Table S4).

### Limitations

Our results are subject to several limitations. Our findings are based on NPs' scope of practice and prescribing authority for nonscheduled legend drugs, as is PrEP. Some laws might not have been included because of the search terms, or exclusion criteria applied. Our results do not account for practice limitations articulated by licensing bodies if those limitations did not appear in state statutes or regulations, or individual collaborative agreements between NPs and supervising providers.

## Discussion and Conclusion

In 2015, over 1 million adults, across three transmission risk groups (gay, bisexual, and other men who have sex with men; heterosexuals; and persons who inject drugs), were estimated to have indications for PrEP use.^3^ A high number and proportion of blacks are estimated to have indications for PrEP but also have very low uptake of PrEP.^3,4^ In 2015, the number of adults with indications for PrEP was greater for blacks than for persons of any other race/ethnicity among gay, bisexual, and other men who have sex with men; and heterosexually active adults; and nearly equal to whites among persons who inject drugs.^3^

In 2020, 1,216,210 people in the United States had PrEP indications, of whom 468,540 were Black people.^4^ However, in 2020, PrEP coverage was only 8.4% for Black people.^4^ Therefore, increasing PrEP access and uptake, for blacks in all transmission risk groups and gay, bisexual, and other men who have sex with men (of all race/ethnicities), is essential in reaching national HIV prevention goals.^3,4^

NPs may help increase PrEP access and uptake for people with PrEP indications, as they play an important role in the primary health care settings that are encouraged to provide the clinical aspects of PrEP.^5,6^ NPs' ability to determine the need for, prescribe, and follow-up patients on PrEP can vary based on both (1) the specificity of the agreements between NPs and supervising physicians and (2) the legal restrictions on nonscheduled drugs prescribing authority.

A recent study found that NPs in states that grant independent scope of practice were 1.4 times more likely to prescribe PrEP than NPs in states that continuously restrict scope of practice.^6^ The least restrictive laws for PrEP access and uptake would allow NPs to practice independently and prescribe legend nonscheduled drugs without restrictions. As of June 8, 2019, 17 jurisdictions had the least restrictive laws and almost half of all NPs worked in a state where both NP practice and prescribing authority are limited.^9^

Over half million persons with indications for PrEP live in a state where both practice and prescribing authority are limited for NPs.^3^ Importantly, four states with the largest estimated number of adults with PrEP indications across the three transmission risk groups (CA, FL, NY, and TX) accounting for 40% of the national total also have restrictive scope of practice laws for NPs.^3^

Increasing PrEP access, uptake, and adherence is a cornerstone of the EHE initiative.^8^ Communities receiving EHE funds are required to implement innovative strategies to increase PrEP prescriptions among persons with indications for PrEP.^8^ NPs may help provide PrEP access and uptake to the >1 million adults who have indications for PrEP in the United States, including Black people and gay, bisexual, and other men who have sex with men (of all race/ethnicities). Policy makers could consider the role that state scope of practice laws plays in potentially limiting NPs' ability to prescribe PrEP.

## Supplementary Material

Supplemental data

Supplemental data

Supplemental data

Supplemental data

## Abbreviations Used

ASTHO: Association of State and Territorial Health Officials
EHE: Ending the HIV Epidemic in the United States
NCSL: National Conference of State Legislatures
NPs: nurse practitioners
PrEP: pre-exposure prophylaxis

## Appendix

### General Practice Authority

(1) Is practice fully independent?
(2) If yes, from day one **or** after a transition period?
(3) If transition period, how long is the transition period?
(4) If yes, other?
(5) Is a relationship with a physician or other health professional required for practice?
(6) If yes, what type of relationship is required (collaborative agreement, protocol, supervision, **and/or** other)?
(7) If supervision, what type is required?
(8) If any, are the protocols at the practice level, state board, or other level?
(9) If any, do collaborative agreements and/or protocols need to be approved by a third party?
(10) If approval needed, what is/are the approval body or bodies?

### Prescribing Authority

(11) Is prescribing authority limited (governed by)?
(12) At the practice level?
(13) By collaborative agreement?
(14) By protocols?
(15) By law?
(16) Other?
(17) Are drugs allowed to be prescribed limited?
(18) If yes, what drugs are limited?
(19) Are prescribing practices for nonscheduled drugs limited?
(20) If yes, what are the practices that are limited?

## References

[1] Fauci AS, Redfield RR, Sigounas G, Weahkee MD, Giroir BP. Ending the HIV epidemic: a plan for the United States. JAMA. 2019;321:844–845, at 844. 30730529 10.1001/jama.2019.1343

[2] HIV Basics PrEP (Pre-exposure prophylaxis). Available at https://www.cdc.gov/hiv/basics/prep.html Accessed December 7, 2021.

[3] Smith D, Van Handel M, Grey J. Estimates of adults with indications for HIV preexposure prophylaxis by jurisdiction, transmission risk group, and race/ethnicity, United States 2015. Ann Epidemiol. 2018;28:850–857, at 854. 29941379 10.1016/j.annepidem.2018.05.003

[4] CDC, NCHHSTP, Atlas. Available at https://gis.cdc.gov/grasp/nchhstpatlas/tables.html Accessed December 7, 2021.

[5] Barnes H, Richards M, McHugh M, et al. Rural and nonrural primary care physician practices increasingly rely on nurse practitioners. Health Affairs. 2018;37:908–914, at 912. 29863933 10.1377/hlthaff.2017.1158 PMC6080248

[6] Carnes N, Zhang J, Gelaude D, et al. Restricting access: a secondary analysis of scope of practice laws and pre-exposure prophylaxis prescribing in the United States, 2017. J Assoc Nurses AIDS Care. 2021 [Epub ahead of print]; DOI: 10.1097/JNC.0000000000000275. PMC9811310 34086636

[7] See for example, West's Revised Code of Washington Annotated 69.41.010 (13) (2019);21 United States Code §812 (2019).

[8] Centers for Disease Control and Prevention. PS20-2010: Integrated HIV Programs for Health Departments to Support Ending the HIV Epidemic in the U.S. Logic Model. Available at https://www.cdc.gov/hiv/funding/announcements/ps20-2010/index.html Accessed December 7, 2021.

[9] Kaiser Family Foundation. Total Number of Nurse Practitioners by Gender. Available at https://www.kff.org/other/state-indicator/total-number-of-nurse-practitioners-by-gender/?currentTimeframe=0&sortModel=%7B%22colId%22:%22Location%22,%22sort%22:%22asc%22%7D Accessed December 7, 2021.

