# Supplementary material for: Mapping Nurse Practitioners' Scope of Practice Laws: A Resource for Evaluating Pre-Exposure Prophylaxis Prescriptions
Source: Health Equity. 2022 Jan 20;6(1):27–31. doi: 10.1089/heq.2021.0113 (PMC8804241; doi:10.1089/heq.2021.0113)
Supplement: Supplemental data [file Suppl_TableS1.docx]

**Supplemental Table 1: Status of Nurse Practitioners’ Scope of Practice (N=51)**

| **Fully Independent (N=30)** | | | **Restricted Practice (N=21)** |
| --- | --- | --- | --- |
| ***Fully Independent (N=27)***   - Alaska - AK - Arizona - AZ - Colorado - CO - Connecticut - CT - Delaware - DE - Hawaii - HI - Idaho - ID - Illinois - IL - Maine - ME - Maryland - MD - Minnesota - MN - Montana - MT - Nebraska - NE - Nevada – NV - New Hampshire – NH - New Mexico- NM - North Dakota - ND - Oklahoma - OK - Oregon – OR - Rhode Island - RI - South Dakota - SD - Utah - UT - Vermont - VT - Virginia – VA - Washington - WA - West Virginia - WV - Wyoming – WY | ***Fully Independent If Not Prescribing (N=2)***   - Kentucky- KY - Massachusetts – MA | ***Fully Independent If Not Prescribing Scheduled Drugs (N=1)***   - Michigan- MI | - Alabama - AL - Arkansas - AR - California - CA - Florida - FL - Georgia - GA - Indiana - IN - Iowa - IA - Kansas - KS - Louisiana - LA - Mississippi - MS - Missouri - MO - New Jersey - NJ - New York - NY - North Carolina - NC - Ohio - OH - Pennsylvania - PA - South Carolina - SC - Tennessee - TN - Texas - TX - Wisconsin – WI - District of Columbia- DC |
