# Supplementary material for: Mapping Nurse Practitioners' Scope of Practice Laws: A Resource for Evaluating Pre-Exposure Prophylaxis Prescriptions
Source: Health Equity. 2022 Jan 20;6(1):27–31. doi: 10.1089/heq.2021.0113 (PMC8804241; doi:10.1089/heq.2021.0113)
Supplement: Supplemental data [file Suppl_TableS2.docx]

**Supplemental Table 2: Variance within the Scope of Practice of Fully Independent Nurse Practitioners**

| **Fully Independent (N=30)** | |
| --- | --- |
| ***From Day One (N=20)***   - Alaska – AK - Arizona – AZ - Colorado – CO - Hawaii – HI - Idaho – ID - Kentucky- KY - Massachusetts - MA - Michigan - MI - Montana – MT - Nevada – NV - New Hampshire – NH - New Mexico - NM - North Dakota – ND - Oklahoma – OK - Oregon – OR - Rhode Island – RI - Utah – UT - Washington – WA - West Virginia – WV - Wyoming – WY | ***After a Transition Period (N=10)***   - Connecticut - CT - Delaware - DE - Illinois - IL - Maine - ME - Maryland - MD - Minnesota - MN - Nebraska – NE - South Dakota - SD - Vermont - VT - Virginia – VA |
