# Supplementary material for: Mapping Nurse Practitioners' Scope of Practice Laws: A Resource for Evaluating Pre-Exposure Prophylaxis Prescriptions
Source: Health Equity. 2022 Jan 20;6(1):27–31. doi: 10.1089/heq.2021.0113 (PMC8804241; doi:10.1089/heq.2021.0113)
Supplement: Supplemental data [file Suppl_TableS3.docx]

**Supplemental Table 3: Status of Nurse Practitioners’ Prescribing Authority (N=51)**

| **Unrestricted Prescribing Authority (N=24)** | **Limited Prescribing Authority (N=27)** |
| --- | --- |
| - Alaska - AK - Delaware - DE - Idaho - ID - Indiana - IN - Iowa - IA - Maine - ME - Maryland - MD - Minnesota - MN - Montana - MT - Nebraska - NE - New Hampshire - NH - New Jersey - NJ - New York - NY - North Carolina - NC - North Dakota - ND - Oregon - OR - Rhode Island - RI - South Carolina - SC - South Dakota - SD - Vermont - VT - Virginia - VA - Washington - WA - Wisconsin – WI - Wyoming - WY | - Alabama - AL - Arizona - AZ - Arkansas - AR - California - CA - Colorado - CO - Connecticut - CT - Florida - FL - Georgia - GA - Hawaii - HI - Illinois - IL - Kansas - KS - Kentucky - KY - Louisiana - LA - Massachusetts - MA - Michigan - MI - Mississippi - MS - Missouri - MO - Nevada - NV - New Mexico - NM - Ohio - OH - ***Oklahoma – OK**** - ***Pennsylvania – PA^#^*** - Tennessee - TN - Texas - TX - Utah - UT - West Virginia - WV - District of Columbia- DC   **** Restricts Prescribing Authority for All Drugs Including Nonscheduled Drugs (i.e. PrEP)*** |

*******Written statement that defines appropriate referral, consultation, and collaboration between the Advanced Practice Registered Nurse, and the supervising physician must be part of the initial application and the renewal application submitted for recognition for prescriptive authority for the Advanced Practice Registered Nurse.

# Written agreement with collaborating physician must include the categories of drugs from which the nurse may prescribe or dispense.
