# Supplementary material for: Mapping Nurse Practitioners' Scope of Practice Laws: A Resource for Evaluating Pre-Exposure Prophylaxis Prescriptions
Source: Health Equity. 2022 Jan 20;6(1):27–31. doi: 10.1089/heq.2021.0113 (PMC8804241; doi:10.1089/heq.2021.0113)
Supplement: Supplemental data [file Suppl_TableS4.docx]

**Supplemental Table 4: Matrix of Nurse Practitioner Scope of Practice and Prescribing Authority**

|  | **Unrestricted Prescribing Authority**  **N=24** | **Restricted Prescribing Authority**  **N=27** |
| --- | --- | --- |
| **Fully Independent**  **N=30** | **N=17**   - Alaska- AK - Delaware- DE - Idaho- ID - Maine- ME - Maryland- MD - Minnesota- MN - Montana- MT - Nebraska- NE - New Hampshire- NH - North Dakota- ND - Oregon- OR - Rhode Island- RI - South Dakota- SD - Vermont- VT - Virginia- VA - Washington- WA - Wyoming- WY | **N=13**   - Arizona- AZ - Colorado-CO - Connecticut- CT - Hawaii- HI - Illinois- IL - Kentucky- KY - Massachusetts- MA - Michigan- MI - Nevada- NV - New Mexico- NM - ***Oklahoma- OK**** - Utah- UT - West Virginia- WV |
| **Restricted Practice**  **N=21** | **N=7**   - Indiana- ID - Iowa- IA - New Jersey- NJ - New York- NY - North Carolina- NC - South Carolina- SC - Wisconsin- WI | **N=14**   - Alabama- AL - Arkansas- AR - California- CA - Florida- FL - Georgia- GA - Kansas- KS - Louisiana- LA - Mississippi- MS - Missouri- MO - Ohio- OH - ***Pennsylvania- PA**** - Tennessee- TN - Texas- TX - District of Columbia- DC |

****Explicitly Restricts Prescribing Authority for All Drugs, Including Non-Scheduled Drugs (i.e. PrEP).***
